# Health-related quality of life as a predictor of mortality in patients on peritoneal dialysis^1^

**DOI:** 10.1590/1518-8345.0786.2687

**Published:** 2016-05-17

**Authors:** Marília Pilotto de Oliveira, Luciana Kusumota, Vanderlei José Haas, Rita de Cássia Helú Mendonça Ribeiro, Sueli Marques, Graziella Allana Serra Alves de Oliveira Oller

**Affiliations:** 2 Doctoral Student, Escola de Enfermagem de Ribeirão Preto, Universidade de São Paulo, PAHO/WHO Collaborating Centre for Nursing Research Development, Ribeirão Preto, SP, Brazil. RN, Hospital das Clínicas, Faculdade de Medicina de Ribeirão Preto, Universidade de São Paulo, Ribeirão Preto, SP, Brazil; 3 Professor, Escola de Enfermagem de Ribeirão Preto, Universidade de São Paulo, PAHO/WHO Collaborating Centre for Nursing Research Development, Ribeirão Preto, SP, Brazil; 4 Professor, Faculdade de Medicina de São José do Rio Preto, São José do Rio Preto, SP, Brazil; 5 Professor, Universidade Federal do Triângulo Mineiro, Uberaba, MG, Brazil; 6 Professor, Escola de Enfermagem de Ribeirão Preto, Universidade de São Paulo, PAHO/WHO Collaborating Centre for Nursing Research Development, Ribeirão Preto, SP, Brazil; 7 Doctoral Student, Escola de Enfermagem de Ribeirão Preto, Universidade de São Paulo, PAHO/WHO Collaborating Centre for Nursing Research Development, Ribeirão Preto, SP, Brazil. Assistant Professor, Universidade Paulista, São José do Rio Preto, SP, Brazil

**Keywords:** Quality of Life, Peritoneal Dialysis, Death, Nursing

## Abstract

**Objective:**

to characterize deaths that occurred, and the association between socio-demographic, clinical, laboratory variables and health-related quality of life and the outcome of death in patients on peritoneal dialysis, over a two year period after an initial assessment.

**Method:**

observational, prospective population study with 82 patients on peritoneal dialysis. The instruments used for the first stage of data collection were the mini-mental state examination, a sociodemographic, economic, clinical and laboratory questionnaire and the Kidney Disease and Quality of Life-Short Form. After two years, data for characterization and occurrence of death in the period were collected. The relative risk of death outcome was calculated through statistical analysis; the risk of death was estimated by the survival Kaplan-Meier curve, and determined predictors of death by the Cox Proportional Hazards Model.

**Results:**

of the 82 original participants, 23 had as an outcome death within two years. The increased risk for the outcome of death was associated with a lower mean score of health-related quality of life in the physical functioning domain.

**Conclusion:**

the worst health-related quality of life in the physical functioning domain, could be considered a predictor of death.

## Introduction

Mortality among patients on dialysis has decreased over time, due mainly to advances in treatment; however, when compared to the general population, mortality is still very high in patients on dialysis. The mortality of patients on peritoneal dialysis (PD) in the United States decreased by 15% between 1993 and 2002, and 35% between 2003 and 2012^(^ 1 ^)^.

Currently, in addition to the interest in increasing the survival time, there is also concern about how these patients experience the years aggregated with advances in treatment.

Peritoneal dialysis provides more flexibility in the treatment of patients with terminal chronic renal disease (CRD), as it can be performed at home by the patient himself and/or his caregiver. Some studies have demonstrated that this dialysis modality generally ensures greater satisfaction with treatment and less impact on the lives of patients, when compared with hemodialysis^(^ 2 ^)^.

Peritoneal dialysis has been used for more than 30 years in Brazil; however, publications on the epidemiology and clinical experience in this type of treatment, as well as the number of patients who opt for it, are incipient^(^ 3 ^-^ 4 ^)^.

The literature has described the losses observed in the *health-related quality of life* (HRQOL) of PD patients, due to CRD and its treatment^(^ 5 ^-^ 6 ^)^.

The construct of quality of life (QOL) has received much attention in recent decades and, although there is no agreed definition, health researchers are interested in the aspects of QOL that are affected by diseases and treatments, therefore QOL assessment in this area is based on a person's self-perception regarding the impact on a number of important aspects and their ability to influence their health. Examples of these clinical and non-clinical aspects are: assessment of general health, physical health, mental/emotional state, social function, sexual function, aspects related to the disease, as well as indirect consequences such as unemployment and financial difficulties that converge to form a construct designated HRQOL^(^ 7 ^-^ 8 ^)^.

The HRQOL has been identified as an important predictor of outcomes in the evolution of treatment, as well as a relevant factor in the choice of treatment by the patient ^(^ 9 ^)^. The low HRQOL scores perceived by patients on dialysis have been reported as predictors of morbidity, hospitalization, and mortality^(^ 10 ^-^ 13 ^)^.

The association between HRQOL and the outcome of death in patients on dialysis has been investigated in other countries, where the worst HRQOL scores have been found to be the main predictors of death^(^ 10 ^,^ 14 ^)^. Recently, in Brazil, this association was investigated in patients on HD and it was verified that the worst HRQOL, particularly in the physical functioning domain, could be considered a predictor of death^(^ 12 ^)^.

In this context, it is necessary to optimize the HRQOL of patients on dialysis, in addition to survival^(^ 3 ^)^.

The analysis of possible associations between HRQOL, socio-demographic, clinical and laboratory aspects, and the outcome of death can be useful in determining targeted areas for the planning of patient care, as well as providing support for further dissemination of this modality between new patients on dialysis. Therefore, this study aimed to characterize the deaths that occurred and the association between socio-demographic, clinical, laboratory, HRQOL and the outcome of death in patients on PD, over a period of two years, after an initial assessment.

## Method

This was an observational, prospective population survey, conducted between 2010 and 2012. It was performed in the two PD services in Ribeirão Preto, São Paulo State.

In the first stage of data collection, which occurred in 2010, the characterization of the 82 patients included in the study and measurement of their HRQOL were conducted. The inclusion criteria were: 18 years or older, receiving treatment by *continuous ambulatory peritoneal dialysis* (*CAPD*) or *automated peritoneal dialysis* (APD) for three months or more, with an intact cognitive state, according to results of the *Mini Mental State Examination (MMSE*)^(^ 15 ^)^, not hospitalized for acute complications or being treated for peritonitis.

After two years, the data on the occurrence of the outcome of death in the period, and the cause, were collected. There was a loss of six patients for follow-up due to: recovery of renal function (two) and county transfer (four). Therefore, of the 82 patients who were interviewed in 2010, 53 remained alive and 23 presented with the outcome of death, which was the target of analysis of the current study.

**Figure 1:**
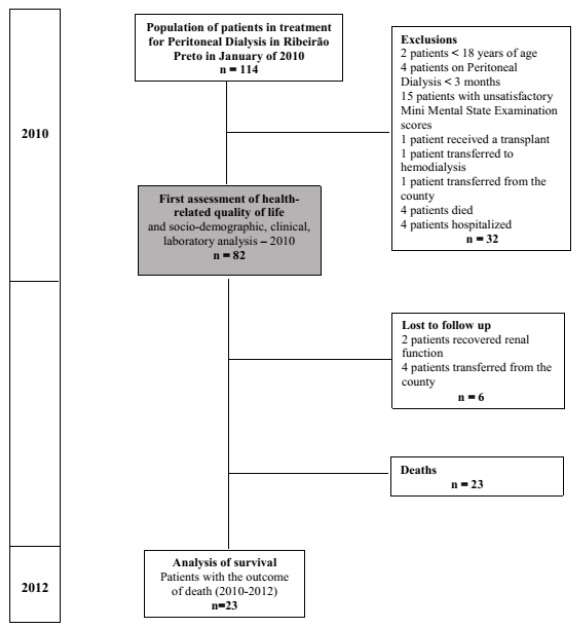
- Flowchart of the steps of the research, selection and maintenance of the patients during the study period

Figure 1 represents the flowchart of the steps of the research, selection and maintenance of the patients during the study period.

The data collection was performed by the researcher, by means of interviews on the day of monthly consultation, from January to March of 2010. Two years later, the data related to deaths in the period, and information about them, were identified through consultation of the records of patients and services.

In the first stage of data collection the instruments used were: MMSE^(^ 15 ^)^ for cognitive assessment; an instrument for socioeconomic and demographic characterization; the terminal CRD and PD adapted and validated for the study, and the *Kidney Disease and Quality of Life-Short Form* (KDQOL-SF^TM)^^(^ 16 ^)^ in the translated version, adapted to the Brazilian culture and available to evaluate the HRQOL^(^ 17 ^)^. The KDQOL-SF^TM^ is an instrument that includes, as a general measure of assessment of the general health of the individual, the *Medical Outcomes Study* (MOS) 36 Item *Short-Form Health Survey* (SF-36), is composed of eight domains: physical functioning, role limitations due to physical health; role limitations due to emotional problems; energy/fatigue; emotional well-being; social functioning; pain; general health. And a multi-item scale with eleven specific dimensions for people with terminal CRD on dialysis, including: symptoms / physical problems, effects of kidney disease on daily life, burden of kidney disease, cognitive function, work status, sexual function, quality of social interactions, and sleep function. The final score ranges from 0 to 100, where zero corresponds to the worst HRQOL and 100 the best HRQOL^(^ 17 ^)^.

Independent variables considered were: sex, age group, number of comorbidities, diabetes mellitus, systemic arterial hypertension (SAH), type of PD, results of laboratory tests (albumin and hemoglobin); the outcome death was considered to be a variable. 

The project was approved by the Ethics Committee of the Ribeirão Preto School of Nursing, University of São Paulo, according to Protocol no. 1451 / 2011. After being invited and agreeing to participate in the study, the patients signed the Terms of Free and Informed Consent, in compliance with Resolution no. 466/12, Guidelines and Regulatory Standards for Research involving human beings of the National Health Council. For the data analysis, the Statistical Package for the Social Sciences (SPSS), version 17.0 was used. The sequential steps of analysis were: descriptive, bivariate and multivariate, and to include independent variables (predictors) in the analytic process, the conceptual criterion was considered based on the theoretical framework in question. First, a descriptive analysis was performed, and the unadjusted relative risk and confidence interval were calculated to estimate the risk of death in the period between 2010 and 2012, according to the predictors investigated in 2010. The Kaplan-Meier survival curves were obtained in order to observe the behavior of the proportion of unadjusted survival of PD patients, according to variables of interest; however, only those with clinical and statistical relevance were presented. For the multivariate analysis, we used the Cox proportional hazards model, verifying the proportionality requirements of atypical risks and values as a prerequisite for use of the regression model. Censoring was defined as patients who did not have the outcome of death, during the period, and were included in the analysis^(^ 18 ^)^. The significance level was 5%.

## Results

The outcome of death was present in 23 (30.3%) patients within two years. The causes of death were: five (21.7%) from sepsis, four (17.4%) from acute myocardial infarction, four (17.4%) with congestive heart failure, two (8.7%) due to acute pulmonary edema, and one (4.3%) for the causes of hyperkalemia, stroke, lung cancer, respiratory failure, and multiple organ failure. For three patients, the information about the cause of death was not obtained.

The survival rate of PD patients was 69.7% over the two-year period of follow up, and the mean survival time in this period was 23.4 months.

This study investigated the HRQOL of the 82 PD patients, by mean of the KDQOL-SF^TM^ in 2010; at that time the physical functioning dimension achieved one of the lowest mean scores of the entire instrument, thus it was the dimension selected to compose the analyses related to death and survival of patients in this study.

In Table 1 the risk of death of patients on PD are presented, according to sociodemographic, clinical, laboratory and HRQOL variables.

**Table 1:** - Risk of death of patients on peritoneal dialysis, in the period of 2010-2012, according to sociodemographic, clinical, laboratory and HRQOL variables in 2010. Ribeirão Preto, Brazil, in 2012

	Survivors		Deaths		Total	RR*	CI 95%	p				
	n=53	%		n=23	%		n=76	%				
Sex												
	Male	21	39.6		11	47.8		32	42.1	1.260	(0.639-2.487)	0.506
	Female	32	60.4		12	52.2		44	57.9			
Age group												
	60 + or	27	50.9		17	73.9		44	57.9	2.061	(0.915-4.640)	0.062
	≤59	26	49.1		6	26.1		32	42.1			
Hypertension												
	Yes	50	94.3		19	82.6		69	90.8	0.482	(0.228-1.017)	0.104
	No	3	5.7		4	17.4		7	9.2			
Diabetes												
	Yes	16	30.2		13	43.5		29	38.2	2.107	(1.065-4.168)	0.030
	No	37	69.8		10	56.5		47	61.8			
Type of dialysis												
	CAPD†	25	47.2		16	69.6		41	53.9	1.951	(0.908-4.194)	0.72
	APD‡	28	52.8		7	30.4		35	46.1			
Albumin												
	Altered	27	50.9		12	52.2		39	51.3	1.035	(0.523-.050)	0.921
	Normal	26	49.1		11	47.8		37	48.7			
Hemoglobin												
	Altered	16	30.2		6	26.1		22	28.9	0.866	(0.394-1.904)	0.717
	Normal	37	69.8		17	73.9		54	71.1			
Comorbitties												
	>4	23	43.9		16	69.6		37	48.7	2.168	(1.008-4.664)	0.036
	<3	30	56.6		7	30.4		39	51.3			
HRQOL dimension§ (Physical functioning)												
	Up to 50	20	37.7		16	69.6		36	47.4	2.540	(1.181-5.461)	0.011
	51 or more	33	62.3		7	30.4		40	52.6			

*Relative Risk † *Continuous ambulatory peritoneal dialysis* ‡ *Automated peritoneal dialysis* § Health-related quality of life

When considering the analysis of independent variables that could be associated with the outcome of death in patients on PD, based on calculating the relative risk with statistical significance, it was verified that patients with diabetes with four or more comorbidities, and those with a mean HRQOL score in the physical functioning dimension with a score of up to 50, had a higher risk of having the outcome of death.

In the survival analysis according to the Kaplan-Meier curves, where the probability of survival in the interval of the study can be visualized according to variables of interest, in the analysis of the *log rank test* did not indicate statistically significant differences for the cumulative proportion of survival between age group, those with hypertension, diabetes mellitus, albumin, hemoglobin, or number of comorbidities. However, for the variables type of PD and the dimension of HRQOL, physical functioning of KDQOL-SF, the *log rank test* indicated statistically significant differences for the cumulative proportion of survival, respectively, between groups of patients on CAPD and APD (p = 0031) as well as between groups of patients with scores up to 50 and 51, and more in the physical functioning dimension (p = 0.011). As indicated in the contingency table (Table 1), patients with 50 points or less, a mean score of HRQOL in the physical functioning dimension had a lower survival, as well as those on CAPD.

With multivariate analysis, and use of the Cox proportional hazards model, it was possible to confirm the HRQOL dimension of physical functioning as a predictor of death. In the regression model presented in Table 2, for every point of increase in the physical functioning dimension, the risk of death decreases by 1.8%, when adjusted for age group, treatment time, type of PD, and number of comorbidities. 

**Table 2:** - Risk of death by Cox proportional hazards, for variables of interest. Ribeirão Preto. SP. Brazil. 2012

	Hazard ratio*	CI 95%	p
Age group	1.429	(0.501-4.074)	0.504
Time of treatment	0.992	(0.971-1.013)	0.459
Type of peritoneal dialysis	2.470	(0.926-6.586)	0.071
Number of comorbities	0.816	(0.272-2.444)	0.716
HRQOL dimension* (Physical functioning)	0.982	(0.968-0.996)	0.014

*Health-related quality of life

The results of survival analysis confirmed the hypothesis that patients with worse scores of HRQOL, specifically in the physical functioning dimension, showed an increased risk for the outcome of death.

## Discussion

The HRQOL has also been investigated as a predictor of outcomes for patients in renal replacement therapy (RRT)^(^ 10 ^-^ 14 ^)^. Particularly in this study, the analysis of HRQOL and other variables of interest was performed, as predictors of the outcome of death for PD patients. The outcome of death was observed in 23 (30.3%) patients within two years after the initial evaluation. It is known that patients with terminal CRD in RRT have a lower survival rate when compared to the general population. Death was also reported as a major cause of discontinuation of dialysis treatment in another study conducted in Brazil with PD patients^(^ 19 ^)^.

Regarding the cause of death, there was a higher frequency of cardiovascular disease and infection, a result that corroborates the findings in other studies that evaluated patients on PD^(^ 10 ^,^ 13 ^)^. There is a risk of unfavorable prognosis associated with cardiovascular disease in these patients, as well as the need to prevent infections, especially peritonitis, which is common in this dialysis modality.

The mean time of survival in PD for patients in this study was 23.4 months. In a survival analysis study conducted in Brazil, considering the initial treatment modality, a lower survival was observed in patients on PD that initiated RRT, who had a mean survival time of 28 months in a three-year follow-up^(^ 20 ^)^.

The risk factors for death, among others, were: initiate dialysis with PD, female, having diabetes as a cause of CRD, being more than 55 years old when initiating treatment ^(^ 20 ^)^. The characteristics of being an older woman and having diabetes were also common among the patients who died in this study.

The survival rate among the PD patients in this study was 69.7% over two years, similar to that found in a multicenter two year follow-up study conducted in Brazil with PD patients, in which the observed survival rate was 70%^(^ 21 ^)^. Similar results were noted in the United States and Canada, in which two-year survival rates were 63.2% and 79.7%, respectively^(^ 22 ^)^. 

In the exploratory analysis of the current study, observed risk factors for death were: diabetes, four or more comorbidities, and a mean score of less than 50 in the physical functioning dimension of the HRQOL. In the Kaplan-Meier curves, patients on CAPD and those with mean HRQOL scores of up to 50, in the physical functioning dimension, showed lower survival times.

It is common for patients with terminal CRD to accumulate comorbidities in addition to CRD throughout life. In addition to the impact in HRQOL observed from the comorbidities, impairment in the lives of patients that accumulate them has been observed in the literature. In the current study, patients with four or more comorbidities had a higher risk of having the outcome of death when compared to those with up to three comorbidities. The greatest risk calculated by the Klan comorbidity index was appointed as a predictor of mortality in a study conducted in Brazil with hemodialysis (HD), patients followed for two years^(^ 12 ^)^. It is noteworthy that patients with cardiovascular disease and diabetes had the lowest survival^(^ 23 ^-^ 24 ^)^.

Diabetes, in particular, proved to be a risk for death in this study. The elderly patients on PD with cardiovascular disease and diabetes had the lowest survival in a study conducted in Brazil^(^ 23 ^)^. In a study of Canadian patients that sought to compare survival between patients on HD and PD, a lower survival was observed in elderly patients with diabetes on PD^(^ 24 ^)^.

The PD treatment can be administered in different ways, either manually or with a cycler to perform the exchange of dialysate, intermittently or continuously, that keeps the abdominal cavity either dry or wet during the day. The clinical condition and the lifestyle of the patient determine the type of PD^(^ 25 ^)^. Each type of PD has advantages and disadvantages, which therefore may impact differently on HRQOL and survival of patients. 

The survival of CAPD patients was worse when compared to those in APD in the survival analysis, according to the Kaplan-Meier curve. However in the multivariate analysis using the Cox proportional hazards, the death risk did not differ in a statistically significant manner between patients on CAPD and APD. In the international literature there were no significant differences in survival of patients who were treated by either type of PD.^(^ 26 ^)^.

Patients who had the outcome of death while receiving PD may not reflect a failure of technique alone, considering that due to the negative selection, many patients using this modality were elderly and also had several comorbidities^(^ 26 ^)^.

A study comparing the two instruments of the Physical Component Summary (PCS), the SF-36 and the SF-12, identified predictors of death in patients on dialysis, highlighted that physical functioning was one of the dimensions that made up the PCS^(^ 11 ^)^. In the current study, patients with a mean score of less than 50 points in the physical functioning dimension of HRQOL had a lower survival rate. Low scores on the PCS were also cited as predictors of lower survival in patients on PD^(^ 13 ^)^.

In a follow-up study of years with patients on HD, a strong association between the lowest mean scores in the three main KDQOL-SF(tm) components and lowest survival was found^(^ 10 ^)^. A worsening in the physical functioning dimension was also observed to be a predictor of lower survival, in a follow-up study conducted for two years with Brazilian patients on HD^(^ 12 ^)^. In a follow-up study of patients on HD observed for four years, a worsening in the summary of the physical and mental components of the SF-36 was noted over time, as well as an association with increased risk of death^(^ 14 ^)^.

The lowest HRQOL scores are an important risk for the outcome of death, so the measurement of HRQOL of RRT in patients cannot be neglected and should be routinely implemented in dialysis clinics and in patient follow-up^(^ 11 ^)^.

In accordance with the PD situation in Brazil, this study identified limitations that may have influenced the presented and discussed findings, but that could not be controlled for purposes of conducting the research. The limited number of participants in the study and different treatment times of the patients must be noted. On the other hand, the performance of this observational and prospective study design for investigation and description of variables that are rare in the Brazilian scientific field is relevant. 

The nurse has an important role in addressing some strategies that can improve HRQOL, and consequently the prognosis of patients on PD, such as: investment in functional rehabilitation and maintenance of patients will certainly impact positively on their lives, improving physical aspects and survival.

## Conclusion

This study enabled us to verify that the lowest HRQOL, specifically in the physical functioning dimension, could be considered a predictor of death. Thus, it is possible that a greater investment by the nurse in the functional rehabilitation and maintenance of patients can assist in improving aspects of HRQOL, especially in dimensions related to physical health, thus having a positive impact on the survival of patients on PD.
